# Precision medicine in type 1 diabetes

**DOI:** 10.1007/s00125-022-05778-3

**Published:** 2022-08-22

**Authors:** Alice L. J. Carr, Carmella Evans-Molina, Richard A. Oram

**Affiliations:** 1 Institute of Biomedical and Clinical Science, University of Exeter Medical School, Exeter, UK; 2 Department of Pediatrics, Indiana University School of Medicine, Indianapolis, IN USA; 3 Department of Medicine, Indiana University School of Medicine, Indianapolis, IN USA; 4 Department of Anatomy, Cell Biology, and Physiology, Indiana University School of Medicine, Indianapolis, IN USA; 5 Department of Biochemistry and Molecular Biology, Indiana University School of Medicine, Indianapolis, IN USA; 6 Center for Diabetes and Metabolic Diseases, Indiana University School of Medicine, Indianapolis, IN USA; 7 Herman B. Wells Center for Pediatric Research, Indiana University School of Medicine, Indianapolis, IN USA; 8 Richard L. Roudebush VA Medical Center, Indianapolis, IN USA

**Keywords:** Continuous glucose monitoring, C-peptide, Genetics, Insulin, Personalised medicine, Precision medicine, Review, Type 1 diabetes

## Abstract

First envisioned by early diabetes clinicians, a person-centred approach to care was an aspirational goal that aimed to match insulin therapy to each individual’s unique requirements. In the 100 years since the discovery of insulin, this goal has evolved to include personalised approaches to type 1 diabetes diagnosis, treatment, prevention and prediction. These advances have been facilitated by the recognition of type 1 diabetes as an autoimmune disease and by advances in our understanding of diabetes pathophysiology, genetics and natural history, which have occurred in parallel with advancements in insulin delivery, glucose monitoring and tools for self-management. In this review, we discuss how these personalised approaches have improved diabetes care and how improved understanding of pathogenesis and human biology might inform precision medicine in the future.

**Graphical abstract:**

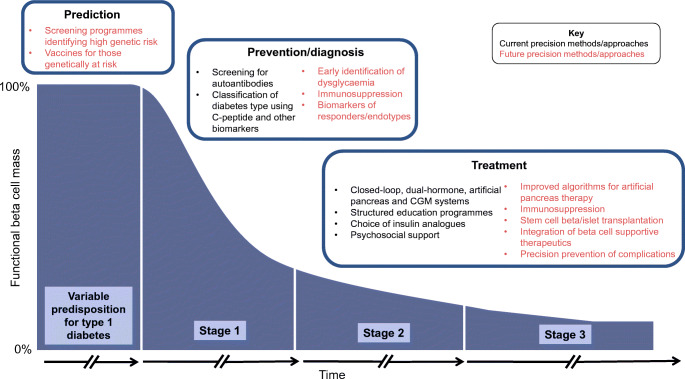

**Supplementary Information:**

The online version contains a slideset of the figures for download available at 10.1007/s00125-022-05778-3.

## Introduction

The year 2022 marks the 100th year since the first patient received insulin. Frederick Banting, Charles Best and James Collip’s transformative discovery of insulin in 1921 has given millions of individuals with type 1 diabetes a second chance at life. Over the ensuing 100 years, type 1 diabetes has evolved from a once inevitable death sentence into a manageable, chronic condition. This evolution has been facilitated by improvements in insulin formulations and insulin delivery, advancements in the convenience, frequency and accuracy of glucose measurement, and the development and application of tools and guidance for lifestyle and dietary management. In parallel, knowledge and understanding of type 1 diabetes pathogenesis have advanced considerably, offering the prospect of therapies that intervene in disease pathogenesis to prevent, reverse or delay the progression of beta cell loss. In this review we describe how advancements in our understanding of type 1 diabetes pathophysiology and treatment have revolutionised clinical care and improved the person-centred approach envisioned by early diabetes clinicians.

The 1920s marked a new era for people living with type 1 diabetes, with insulin injections effectively preventing death from severe insulin deficiency. However, with little understanding of the pathophysiology of diabetes development and no differentiation of disease ‘subtypes’, standards of care remained largely the same for all patients diagnosed with diabetes. Early into this post-insulin era, it was recognised that diabetes is a chronic illness and that treatment would involve the lifelong combination of insulin regimens with diet, exercise and infection protocols [1]. These medical insights were disseminated to both patients and clinicians in manuals on diabetes. One of the first and most detailed manuals to incorporate insulin treatment was developed by Elliot Joslin in 1924 [2]. This manual, which encompassed all knowledge required by those living with diabetes and by their clinicians, covered urinalysis using Benedict’s test for glucose monitoring, administration of insulin, nutritional statistics for a variety of foods, and information on how to treat diabetes with insulin and diet. Joslin was prescient in recognising that treatment regimens should be adjusted to an individual’s needs, stating in 1924 that ‘The treatment of a patient with diabetes lasts through life. Treatment therefore must be adjusted to the condition of the patient and should be so arranged that it can be continued for years, not only without harm, but with as little annoyance and interference with the daily routine as is possible. Consequently, the patient must be taught the nature of his disease and how to conquer it’ [1, 2]. Arguably, Joslin recognised the need for personalised diabetes treatments and the empowerment of those affected. He realised that patients must be taught the tools for self-management to both prolong life and improve the quality of life for those living with diabetes. This idea of a person-centred approach to care expanded quickly into a variety of treatment regimens.

An in-depth knowledge of type 1 diabetes pathogenesis is critical to understanding how precision medicine may apply to type 1 diabetes. In the early 1930s it was noticed that people with diabetes responded differently to insulin, enabling the differentiation between insulin-insufficient and insulin-sensitive subgroups [3, 4]. However, it was not until the 1950s that this observation was confirmed using the first insulin assays, which enabled quantification of circulating insulin in humans [5–7]. From this point, different types of diabetes were considered, but the aetiological basis of the insulin-deficient disease type was not identified as autoimmune in origin until later, with Willy Gepts reporting evidence of immunological infiltrates in the pancreases of newly diagnosed children with diabetes in 1965 [8], which was reinforced by the identification of islet cell autoantibodies by Franco Bottazzo in 1974 [9].

These discoveries formed the foundation of our contemporary understanding of the pathophysiology of type 1 diabetes. The scientific community was quick to accept this paradigm shift, which led to huge advancements in our understanding of the underlying aetiology of type 1 diabetes within the space of a few years. Coincident with these discoveries, clinical observations of familial inheritance of diabetes led to the proposal in the 1950s of a partial genetic basis of diabetes development [10]. Twin studies in children and young adults in the late 1960s and early 1970s reported around 50% concordance of diabetes in monozygotic pairs (presumed to be type 1 diabetes) compared with >90% concordance of diabetes in those diagnosed at older ages (presumed to be type 2 diabetes) [11–13]. In addition to these findings, descriptions of the critical role of HLA antigen-presentation genes in the transplant setting led to the association of these genes with autoimmune diseases [13, 14]. Identification of HLA associations, combined with the discovery of islet cell autoantibodies, established that these genes transmitted the tendency to develop type 1 diabetes, but not the disease itself [14]. These findings were summarised by George Eisenbarth in 1986 in the widely adopted Eisenbarth model, which outlined that genetically predisposed individuals encounter a hypothetical triggering event that begins a process of autoimmune-mediated progressive beta cell destruction leading to insulin deficiency [15].

**Fig. 1:**
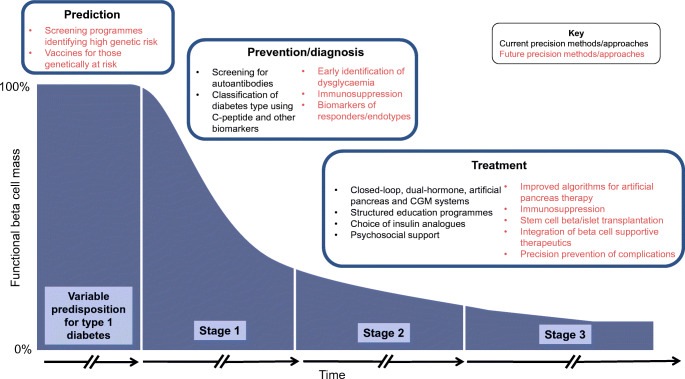
The Eisenbarth model continues to inform strategies for disease prevention and, more recently, precision medicine approaches. Using its most up-to-date form, which describes the stages of type 1 diabetes proposed by Insel and colleagues [16], this figure addresses precision medicine approaches that are, or could be, used at each stage of the model. Beginning in the predisposition phase, we see a future of precision prediction in the form of genetic screening programmes. In stage 1 disease, where autoimmunity begins, and entering into stage 2, current precision prevention options are limited. Screening for autoantibodies in those at high risk is a current helpful option for identifying early disease, with ongoing and future efforts focusing on better identification of these stages and early intervention therapeutics. Around diagnosis, current methods for the precise classification of type 1 diabetes, such as C-peptide measurements and classification models that use a combination of biomarkers, can enable the correct application of precision treatment in type 1 diabetes. In stage 3 overt diabetes, a number of therapies, including advanced technology and education programmes, are already employed in clinical care, with immune and stem cell replacement therapies on the horizon. This figure is available as part of a downloadable slideset

The Eisenbarth model continues to inform strategies for disease prevention and, more recently, precision medicine approaches. The model was updated by Insel and colleagues in 2015 [16] based on a landmark meta-analysis of several birth cohorts which showed that >80% of children who develop two or more islet-specific autoantibodies progress to type 1 diabetes by the age of 20 [17]. It is now recognised that there are three distinct stages of type 1 diabetes that precede clinical diagnosis: stage 1, when islet autoimmunity is measurable by the presence of multiple autoantibodies; stage 2, when there is measurable dysglycaemia; and stage 3, when glucose abnormalities fulfil criteria for clinical diagnosis of diabetes. Summarised in Fig. 1, these three stages have each seen an expansion of increasingly precise approaches encompassing the prediction, prevention, diagnosis and treatment of type 1 diabetes. Individualised prediction is enabling the early diagnosis and prevention of stage 2 diabetes progression, and for those with established stage 3 diabetes there are a multitude of approaches that can be tailored in order to optimise care for the individual, with many more precise approaches, methods and treatments on the horizon.

There is momentum in the field of diabetes to take advantage of ‘recent, rapid scientific advances in our ability to measure and characterise human variation through (1) assessment of the genetic and metabolic state, (2) leveraging data to inform disease categories, and (3) science-guided preventive and treatment decisions tailored to specific pathological conditions’ [18]. The ADA and EASD have partnered to assess the current state of precision medicine in diabetes through a series of systematic reviews across diabetes types, with the aim of understanding the role of precision medicine in diagnosis, subcategorisation, prevention and therapy. The bedrock of good clinical care relies on the human- and person-centred approach advocated by Joslin [2]; however, there are opportunities to take advantage of the increasing understanding of type 1 diabetes pathogenesis to better intervene. Throughout this review we discuss the current diagnostic, treatment and management strategies for people with type 1 diabetes and, with forethought, discuss how the concept of precision medicine can be applied to type 1 diabetes.

## Prediction

Identification of longitudinal biomarkers in the form of islet-specific autoantibodies in large studies of at-risk individuals (either from genetically high-risk infants from birth or first-degree relatives of people with type 1 diabetes [19]) has increased our understanding of the progression to type 1 diabetes and improved the prediction of future type 1 diabetes development. Historically, the at-risk population was identified using HLA typing of type 1 diabetes risk-associated HLA alleles (*HLA-DR3-DQ2* and/or *HLA-DR4-DQ8*, with avoidance of strong protective alleles such as *HLA-DR15-DQ6*) or by identifying infants or adults at risk because of an affected family member [20]. Recent advances in genome-wide association studies and the identification of numerous common variants associated with type 1 diabetes have facilitated the combination of HLA and non-HLA genetic risk into polygenic or genetic risk scores that can be used to aid prediction and/or classification of disease. These technological advances allow for the possibility of performing cheap and efficient genetic screening at birth to identify individuals at risk for developing type 1 diabetes [21, 22]. The increasing integration of genomics into healthcare means it is realistic that, in the future, type 1 diabetes genetic risk may be measurable from birth.

While genetics can identify at-risk individuals, the majority of those classified as ‘high risk’ will probably not develop type 1 diabetes because of the relatively low background prevalence of this disease [23]. Islet-specific autoantibodies are a more specific measure of the development of autoimmunity, and the presence of islet autoantibodies forms the basis of the recently revised type 1 diabetes staging paradigm [16]. Combined analysis of large screening studies may allow for the targeted measurement of islet-specific autoantibodies at key time points during childhood to provide maximum sensitivity and specificity for identifying future type 1 diabetes cases, possibly by integrating screening with other early life healthcare visits [24, 25]. The major biomarkers currently used in predicting future type 1 diabetes development include genetics, age, number, types and titres of autoantibodies and age at which these appear, dysglycaemia and C-peptide levels. These markers can be used individually but provide more predictive power when used in combination [26]. In the future, the increasing availability of genetic information, combined with the proven ability of autoantibody screening to identify early-stage type 1 diabetes, may lead to an era of precision prediction in which we are able to predict type 1 diabetes and intercept before and prevent or delay disease onset.

Many groups are working to improve the precision prediction of type 1 diabetes using novel biomarker and ‘omics’ approaches [27], including advanced omic, single cell and advanced imaging analysis of pancreatic tissue from organ donors with autoantibody positivity and established type 1 diabetes [28–31]. We are now able to study the complex environmental, metabolomic, virome, molecular and microbiome associations in type 1 diabetes progression. A large number of association studies have highlighted the complex interplay between immune abnormalities, genetics and the environment [32–34]. We now have more markers of beta cell stress and dysfunction and increasing evidence of the complex interplay between the environment, beta cells and the immune system. It is possible that these detailed molecular approaches and the application of novel computational approaches that are better able to integrate multiple features may aid with prediction over and beyond current strategies. It is equally important and likely that further mechanistic insights from these approaches may help identify targets for intervention.

## Prevention

Recognition of type 1 diabetes as an autoimmune disease led to attempts at treating the underlying pathogenesis with immunotherapy. Clinical trials that aimed to prevent the progression of early diabetes initially used steroids, such as prednisone [35], in combination with azathioprine [36], anti-thymocyte globulin [37] and ciclosporin [38]. Recently, clinical trials have focused on more selective immune agents, such as the anti-CD3 antibody teplizumab [39] and agents thought to act directly on beta cells (e.g. verapamil) [40, 41]. There have been some recent notable successes of agents, including rituximab [42], teplizumab [43, 44], golimumab [45] and anti-thymocyte globulin [46], tested closer to the onset of stage 3 diabetes. However, none of these agents has led to durable disease remission, and these successes have been against the backdrop of several unsuccessful trials [47, 48]. The findings suggest that there may be irremediable loss of beta cell mass and function after the onset of stage 3 diabetes. To address whether earlier intervention may be more efficacious, teplizumab was tested as a single 14-day course in individuals with two or more autoantibodies and dysglycaemia. In this context, teplizumab delayed the onset of stage 3 disease by a median of 32.5 months [39]. Teplizumab is currently under consideration by the US Food and Drug Administration as the first potential disease-modifying therapy in diabetes, following nearly three decades of preclinical and clinical studies.

While the teplizumab trial in stage 2 disease showed that earlier intervention is a promising approach and that it is possible to delay the onset of clinical disease in some high-risk individuals, there was still considerable heterogeneity in response noted among trial participants. These findings raise the possibility that there may be subgroups of individuals who require different treatment approaches and that heterogeneity in disease progression may be driven by underlying differences in pathophysiology or endotypes. A disease endotype is broadly defined as a subtype of disease originating from a distinct functional or pathobiological mechanism that can be addressed therapeutically [49]. This concept was pioneered in the field of asthma, where distinct endotypes have been defined and targeted therapeutically [50]. However, it is currently not clear whether individuals with type 1 diabetes are ‘more similar than they are different’ and require similar disease-modifying treatments or whether factors such as islet autoimmunity, age at diagnosis and immune phenotype will lead to distinct interventions. Tailored treatments would greatly benefit patients; however, further subdivisions of type 1 diabetes would risk reducing the market for pharmaceutical companies, which already struggle to see a large enough market to invest significantly in type 1 diabetes research.

Notwithstanding this controversy, several recent observations hold promise in identifying type 1 diabetes endotypes. It is well accepted that children and adults exhibit differences in disease progression [51–53], with children having a higher risk of developing diabetes and a more accelerated rate of progression from seroconversion to stage 3 diabetes [17]. Along these lines, there are important differences in islet immune cell infiltrates and proinsulin processing, which are correlative with age at diagnosis [54, 55]. In addition, evidence from multiple birth cohort studies suggest that progression from first autoantibody development may differ by age of onset. Antibody specificity and background genetics have yet to be directly connected to post-diagnosis progression [56]. Furthermore, a very recent analysis by Achenbach and colleagues identified multiple variables that classified young type 1 diabetes patients into seven islet autoantibody-positive and three islet autoantibody-negative subgroups. These subgroups demonstrated substantial differences in pathogenic and prognostic outcomes, which could have therapeutic relevance [57]. Machine learning approaches have also been helpful in identifying autoantibody and disease trajectories [58]. An important future aspiration will be to design experiments that further investigate the mechanisms of possible age-influenced variation in progression to diabetes, pathogenesis at a tissue level, immune phenotype and progression of beta cell loss post diagnosis.

## Diagnosis

The correct classification of diabetes subtype is crucial for the correct application of precision treatment in type 1 diabetes and for the investigation of diabetes pathogenesis. There is considerable evidence of misclassification of type 1 diabetes as type 2 diabetes, and misclassification of monogenic diabetes and type 2 diabetes as type 1 diabetes [59, 60]. A correct diagnosis is important to determine the appropriate treatment, with type 1 diabetes requiring physiological doses of insulin replacement to avoid acute life-threatening complications such as diabetic ketoacidosis. Up to one in three adults with type 1 diabetes are initially diagnosed as having type 2 diabetes [60]. Thus, it is apparent that historical approaches to classification have been unable to provide simple criteria to aid in diagnosis and there is room to use more precise methods of classification. Clinical features are predominately used for classification of diabetes type, with age at diagnosis and BMI having evidence of clinical utility at onset [61]. However, features frequently overlap in adults diagnosed with diabetes, and the high prevalence of type 2 diabetes adds to the difficulty of confirming a diagnosis of type 1 diabetes in adults. Islet autoantibodies can assist in classification, and recent guidance from the ADA and EASD recommend islet autoantibody testing at diagnosis in all adults with clinically suspected type 1 diabetes [62]. More recently, type 1 diabetes genetic risk scores have also been shown to assist in discriminating between type 1, type 2 and other forms of diabetes in research settings [63, 64]. Recent work has shown that these clinical features and biomarkers are most discriminative of diabetes type when combined and modelled as continuous variables in diagnostic models [61, 63, 65].

Rapid progression to insulin deficiency, a major feature of type 1 diabetes, determines treatment and can be used to aid in classification. A marker of endogenous insulin secretion is the level of serum or urine C-peptide, which is co-secreted in equimolar amounts with insulin and has little assay cross-reactivity with exogenous insulin or proinsulin [66, 67]. Severe insulin deficiency not only is a biomarker of type 1 diabetes but also, by definition, indicates a need for insulin replacement, thereby linking treatment to pathogenesis [68]. C-peptide testing in those with clinically diagnosed type 1 diabetes can lead to reclassification and insulin withdrawal [59]. In addition, C-peptide measured within the first few years of diagnosis may be useful in confirming type 1 diabetes if results indicate severe insulin deficiency (e.g. fasting level <80 pmol/l or post-meal level <200 pmol/l [68]), as those with either type 2 diabetes or monogenic diabetes almost always have C-peptide levels above these cut-offs. However, C-peptide levels at diagnosis of type 1 diabetes can overlap with those observed in other diabetes types. Instead, the progressive trajectory of C-peptide loss over the immediate years post diagnosis most clearly separates type 1 diabetes from type 2 diabetes, and the utility of C-peptide levels in discriminating type 1 diabetes is greatest 3–5 years post diagnosis [68]. Recent progress in the ability to measure C-peptide in clinical and remote settings [69–72] has facilitated the integration of C-peptide measurement into national and international diabetes guidelines [62].

## Treatment

Gradual improvements in the formula and delivery of insulin have allowed for significant steps forward in the ability to personalise insulin therapy. Although lifesaving, the insulin preparations of the 1920s were basic and the glucose-lowering effects lasted for only 6 h, thus requiring multiple injections throughout the day. Longer-acting insulins were developed in 1936 through combination of insulin with protamine and then zinc [73, 74]. The discovery of the sequence and structure of insulin, the synthesis of the first synthetic human insulin and the emergence of recombinant DNA technology led to the manufacture of insulins with modifiable properties. These advances eventually gave rise to analogue insulins, which dominate the market today and allow for optimisation of absorption rate, time to peak and duration of action depending on their design. The landmark DCCT was published in 1993 and demonstrated the benefit of intensive insulin therapy and tight glycaemic control for the prevention of microvascular complications [75, 76]. Thus, newer insulin formulations with optimised pharmacokinetics were a welcome addition, as ‘tight glucose control’ became the goal for all individuals in the post-DCCT era. In current practice, clinicians and patients can choose from a range of insulins that can be employed in various regimens to suit an individual’s needs and lifestyle.

The pace of development of additional tools to aid in diabetes management has been rapid since the end of the DCCT. Flash glucose monitoring and continuous glucose monitoring (CGM) involve sensors that measure glucose levels in interstitial fluid every 5–15 min, providing more detailed, daily insights into glucose control beyond that of the 3- to 4-month estimate HbA_1c_ provides. Traditional CGM displays trends and data automatically to the user, while flash CGM requires the user to swipe a sensor with a reader to display blood glucose data. Both methods provide several quantitative measures such as glycaemic variability and time spent above (hyperglycaemia), below (hypoglycaemia) and within clinically defined glucose ranges. Unlike HbA_1c_ measurements and self-monitoring of blood glucose, flash and CGM technologies enable the communication of real-time glucose values, trends and glycaemic variability. These individualised evaluations have been shown to improve HbA_1c_ levels, decrease the time spent in hyperglycaemia and hypoglycaemia and reduce the risk of severe hypoglycaemia [77–80], while also improving quality of life [81]. Adoption of these technologies as standard of care for all patients with type 1 diabetes, as proposed in the recent updates to the UK’s National Institute for Health and Care Excellence (NICE) guidelines [82, 83], represents a much-needed shift toward viewing technology as an integral part of diabetes management. Although HbA_1c_ measurement remains the most robust and validated measurement associated with chronic diabetes complications, insights from studies using CGM suggest that HbA_1c_ is unsuitable for determining short-term glycaemic changes accurately [84–86]. Recent efforts to examine the relationship between CGM-derived time within target glucose range and long-term complications are providing a basis for glycaemic targets for newer glucose monitoring technologies [62, 87].

In addition to improvements in glucose monitoring, the last decade has seen rapid improvements in insulin delivery systems. Continuous subcutaneous insulin infusion systems have demonstrated a small but significant benefit for glycaemic control over that of the traditional multiple daily injection method [88]. However, despite these advances in insulin analogues and delivery systems and glucose sensors, many people with type 1 diabetes still do not achieve glycaemic targets. More recent advances in insulin delivery systems and their integration with CGM technology has enabled automated ongoing adjustment of insulin delivery to optimise glycaemic control throughout the day and night. These ‘closed-loop’ and artificial pancreas systems have been evaluated in children and adolescents and demonstrate improved glucose control and reduced risk of hypoglycaemic events [89–96], even compared with sensor-augmented insulin pumps [97].

Although insulin replacement is essential, it is important to recognise the role that education strategies have in enabling precision treatment in type 1 diabetes. The first of these individualised treatments was the diet regimen developed by Robert Lawrence in 1925 [98]. Lawrence’s ‘line ration scheme’ was designed to be flexible for patients and manageable for clinicians and remained engrained in care as late as the 1950s [99]. Today, this education scheme has evolved into the well-established Dose Adjustment for Normal Eating (DAFNE) programme, which was developed originally in the 1990s in Germany [100] and which was endorsed by the UK NHS [101]. For adults, this programme is an educational tool to enable individuals to understand the carbohydrate content of foods and the correct insulin dosing and apply this to their lifestyle. In addition, the programme covers insulin management during exercise, illness and social activities. It is recognised that patients and clinicians need more in-depth educational strategies that cover the management of behavioural aspects associated with type 1 diabetes, most notably exercise. People with type 1 diabetes face several barriers to exercise; however, ‘lack of knowledge’ is one of the most expressed concerns [102]. Employing educational strategies is crucial in enabling the personalised treatment of type 1 diabetes by allowing patients to drive the management of their disease.

Even with the availability of optimised insulins, new technologies for insulin delivery and glucose monitoring and improved tools for self-management, it is acknowledged that care may differ across a person’s lifespan. This is reflected in recent statements surrounding the individualisation of glycaemic targets. It has been rightfully proposed that the glycaemic target ‘should be individualised considering factors that include duration of diabetes, age and life expectancy, comorbid conditions, known cardiovascular disease or advanced microvascular complications, impaired awareness of hypoglycaemia (IAH) and other individual considerations, and it may change over time’, emphasising that this goal should be achieved in conjunction with an understanding of a person’s psychosocial needs and a reduction in diabetes distress [62]. In addition, specific glycaemic targets are recommended at certain life stages. In particular, women with type 1 diabetes are supported to achieve blood glucose ranges close to those seen in pregnant women without diabetes (HbA_1c_ ≤48 mmol/mol [≤6.5%]) in addition to focused pre- and postprandial glucose targets, in order to reduce the risk of serious adverse pregnancy outcomes [62, 103, 104]. In older adults, safety of insulin use takes precedence, because of their increased vulnerability to hypoglycaemia, with targets based on functional status and life expectancy and adjusted to minimise the occurrence of hypoglycaemic events [62].

Risk of hypoglycaemia is perhaps the largest barrier to intensive diabetes control and is often reported as having a profound impact on quality of life and diabetes self-care behaviours [105, 106]. Additionally, it is possible that fear of hypoglycaemia is a driver of glycaemic variability and suboptimal glucose control. Although there is evidence that closed-loop insulin therapy is beneficial for glycaemic control, with growing evidence that these benefits extend psychosocially [107, 108], some challenges remain. Exercise presents a particular challenge in closed-loop therapy because of the complex glucose physiology that occurs during exercise, with increased glucose turnover and distinct hormonal and metabolic responses to different forms of exercise [109, 110]. Compounded by the lag time of current glucose sensing [111, 112], closed-loop systems that manage exercise without the risk of hypoglycaemia [109, 113, 114] have not yet been achieved.

It is possible that C-peptide measurement may have a role beyond disease classification in precision clinical care, especially in identifying those most likely to achieve restrictive glycaemic targets. Much as behavioural factors influence glucose levels, the biological factor of preserved endogenous insulin also plays a crucial role in glucose control; however, it is not always considered in clinical care. Numerous studies of endogenous insulin production in people with type 1 diabetes highlight the variation in absolute levels of C-peptide both at diagnosis and in long-duration type 1 diabetes [51–53, 115–118]. Additionally, there is heterogeneity of normal development and endowment of beta cells, in the rate of autoimmune destruction of beta cells and in whether autoimmune destruction progresses to complete loss of insulin-producing beta cells [51–55, 115–122]. There is longstanding and emerging evidence that the amount of persistent endogenous insulin a person with type 1 diabetes maintains influences their glycaemic control, risk of hypoglycaemia and risk of long-term complications across the duration of disease [75, 76, 123–130]. Although the key and initial analysis of the DCCT demonstrated the benefit of intensive insulin therapy and tight glycaemic control for the prevention of long-term diabetes complications [75, 76], it came at the cost of higher risk of severe hypoglycaemia (self-reported) in those who received intensive insulin therapy, a barrier to intensive insulin therapy that remains today despite improvements in insulin formulations. However, those who retained the ability to secrete higher levels of C-peptide in response to a stimulus demonstrated a significant reduction in the risk of severe hypoglycaemia in addition to decreased retinopathy and nephropathy progression [75, 76, 123]. Increasing numbers of observational studies support these findings [125–130], with the evidence for persistent endogenous insulin reducing hypoglycaemic risk being most apparent in the setting of islet transplantation, where those at high hypoglycaemia risk commonly have a dramatic reduction of this risk with even modest levels of graft function post transplant. Additionally, the increased use and availability of flash glucose monitoring and CGM has highlighted that there are similar benefits for glycaemic variability and control of persistent C-peptide levels over all durations of diabetes [126, 127, 131]. Routine measurement of C-peptide could aid in understanding the differences in glucose patterns between individuals, regardless of diabetes management behaviours. Incorporation of C-peptide measurements into the standard of care could be an effective approach to supporting newly diagnosed patients by enabling the personalisation of care from the point of diagnosis, which could be expanded across the duration of diabetes. The importance of maintaining C-peptide levels also underlies clinical trial efforts focused on the preservation of beta cell function in those with and at risk of diabetes.

Finally, it is important to recognise that type 1 diabetes is not just a disease of beta cells, as beta cell destruction impacts paracrine interactions within the islet, leading to impairments in the normal secretory patterns of other islet hormones that are critical for glucose homeostasis. In the future, it is possible that the increasing ease with which C-peptide and other islet-associated and glucose-regulating hormones can be measured may allow a more accurate description of an individual’s ability to buffer changes in blood glucose. Moreover, it is possible that the integration of other hormones, such as glucagon, into dual-hormone systems will allow for better management of activities such as exercise that have a high risk for hypoglycaemia [132]. These insights, combined with precision glucose measurements from CGM, will contribute to disease management in terms of lifestyle changes, additional non-insulin medications, and choice of monitoring and insulin administration.

Contemporary diabetes technologies could be considered a gateway for precision medicine in type 1 diabetes, as they enable treatment to be continually adjusted to the condition of the patient, just as Joslin had hoped. However, a number of barriers remain. New technologies are not accessible to everyone because of their cost. This global disparity in the availability of therapies is one of the main barriers to enabling precision treatment in type 1 diabetes. While closed-loop systems provide significant improvements in insulin delivery and glucose monitoring, thus improving glycaemic control and reducing the daily burden of living with diabetes, there are ongoing challenges related to their implementation and they are not yet able to provide an ‘attach and forget’ solution. Furthermore, a diagnosis of diabetes still imposes additional responsibilities and requires planning and self-monitoring. Such a marked readjustment of daily life is inevitably physically and psychologically draining [133]. Depression levels among adults with type 1 diabetes are higher than in the general population [134]. Distinct from depression, diabetes distress [135, 136] is also common in diabetes [137] and is a product of emotional adjustment to the demands of diabetes. Diabetes distress has been found to be significantly associated with higher HbA_1c_ levels [138], with a recent study demonstrating that this was pronounced in youth of lower socioeconomic status and/or racial and ethnic minority youth [139]. Although there are established measures of diabetes distress, including the Problem Areas in Diabetes scale [140] and the Diabetes Distress Scale [141], these emotional issues are frequently not integrated into care. The recognition and understanding of emotional issues in diabetes care is a crucial step towards a person-centred and collaborative approach to care [133]. The recently updated ADA Standards of Medical Care encourage providers to assess symptoms of diabetes distress, depression, anxiety, disordered eating and cognitive capacities using appropriate standardised and validated tools at the initial visit, at periodic intervals and when there is a change in disease, treatment or life circumstance [142]. Integrating tailored education and professional counselling with standard glucose and well-being metrics may improve the precision of clinical decision making and could aid in predicting future emotional crises [18].

## Summary

**Fig. 2:**
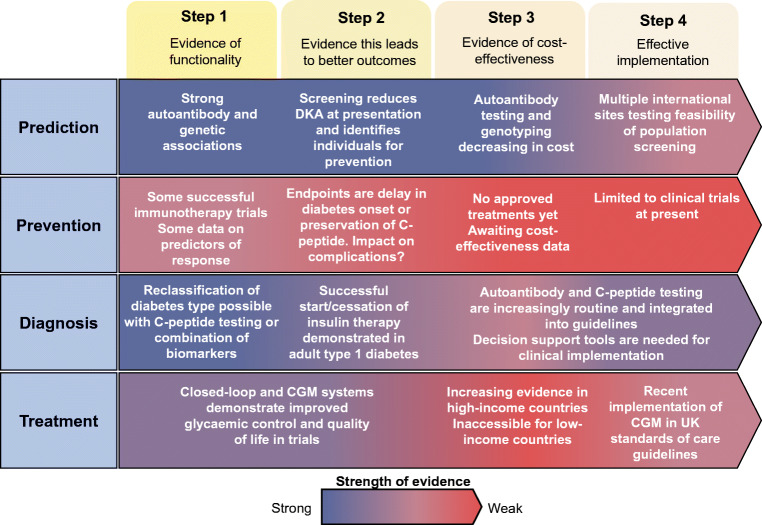
Roadmap for precision medicine in type 1 diabetes across the aspects of prediction, prevention, diagnosis and treatment. Steps 1–4 describe the stages of discovery, validation and implementation required for successful precision medicine approaches. The colour scale depicts the current strength of evidence for each of these steps and highlights gaps that could be targeted in the future. DKA, diabetic ketoacidosis. This figure is available as part of a downloadable slideset

Significant progress has been made in the personalisation of type 1 diabetes treatment since the discovery of insulin in 1922. These improvements include technological advancements in insulin delivery, marked advances in glucose monitoring and the recognition that these technical advances need to be accompanied by personal and psychosocial support for people with type 1 diabetes. Defining the aetiopathogenesis of type 1 diabetes as a complex autoimmune disease, as summarised by the Eisenbarth model nearly 40 years ago, has opened up the possibility of better prediction, diagnosis and, potentially in the future, prevention of type 1 diabetes. Recently, Florez and Pearson proposed a roadmap to achieve pharmacological precision medicine in monogenic and type 2 diabetes [143]. Inspired by this construct, Fig. 2 highlights a similar approach in type 1 diabetes. Here, we outline a roadmap for precision medicine in type 1 diabetes across the aspects of prediction, prevention, diagnosis and treatment, highlighting gaps that could be targeted in the future. We are facing a future of increasingly detailed omics techniques and real-time metabolic monitoring that can describe human biology and disease pathogenesis in ever more detail. We hope that improved prediction and understanding of type 1 diabetes through these methods will ultimately lead to a better understanding of variation in type 1 diabetes pathogenesis and improved disease-modifying treatments and biological interventions that can prevent, stop or reverse type 1 diabetes pathogenesis.

## Supplementary information

Slideset of figures (PPTX 491 kb)

## Abbreviations

CGM: Continuous glucose monitoring
NICE: National Institute for Health and Care Excellence
