# Supplementary material for: Precision medicine in type 1 diabetes
Source: Diabetologia. 2022 Aug 22;65(11):1854–66. doi: 10.1007/s00125-022-05778-3 (PMC9522741; doi:10.1007/s00125-022-05778-3)
Supplement: Supplementary file 1 — (PPTX 491 kb) [file 125_2022_5778_MOESM1_ESM.pptx]

## Slide 1
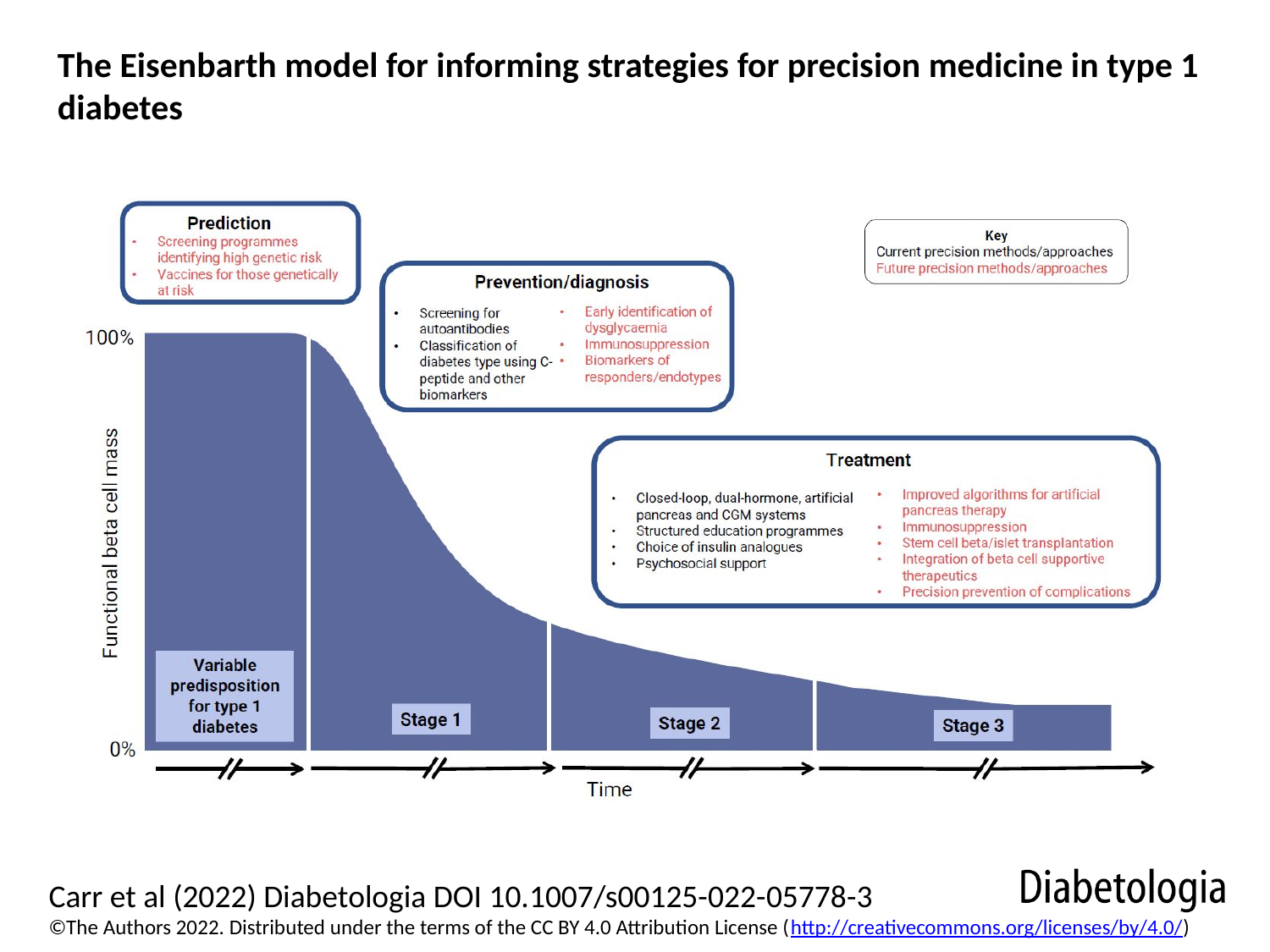

The Eisenbarth model for informing strategies for precision medicine in type 1 diabetes
Carr et al (2022) Diabetologia DOI 10.1007/s00125-022-05778-3
©The Authors 2022. Distributed under the terms of the CC BY 4.0 Attribution License (http://creativecommons.org/licenses/by/4.0/)

## Slide 2
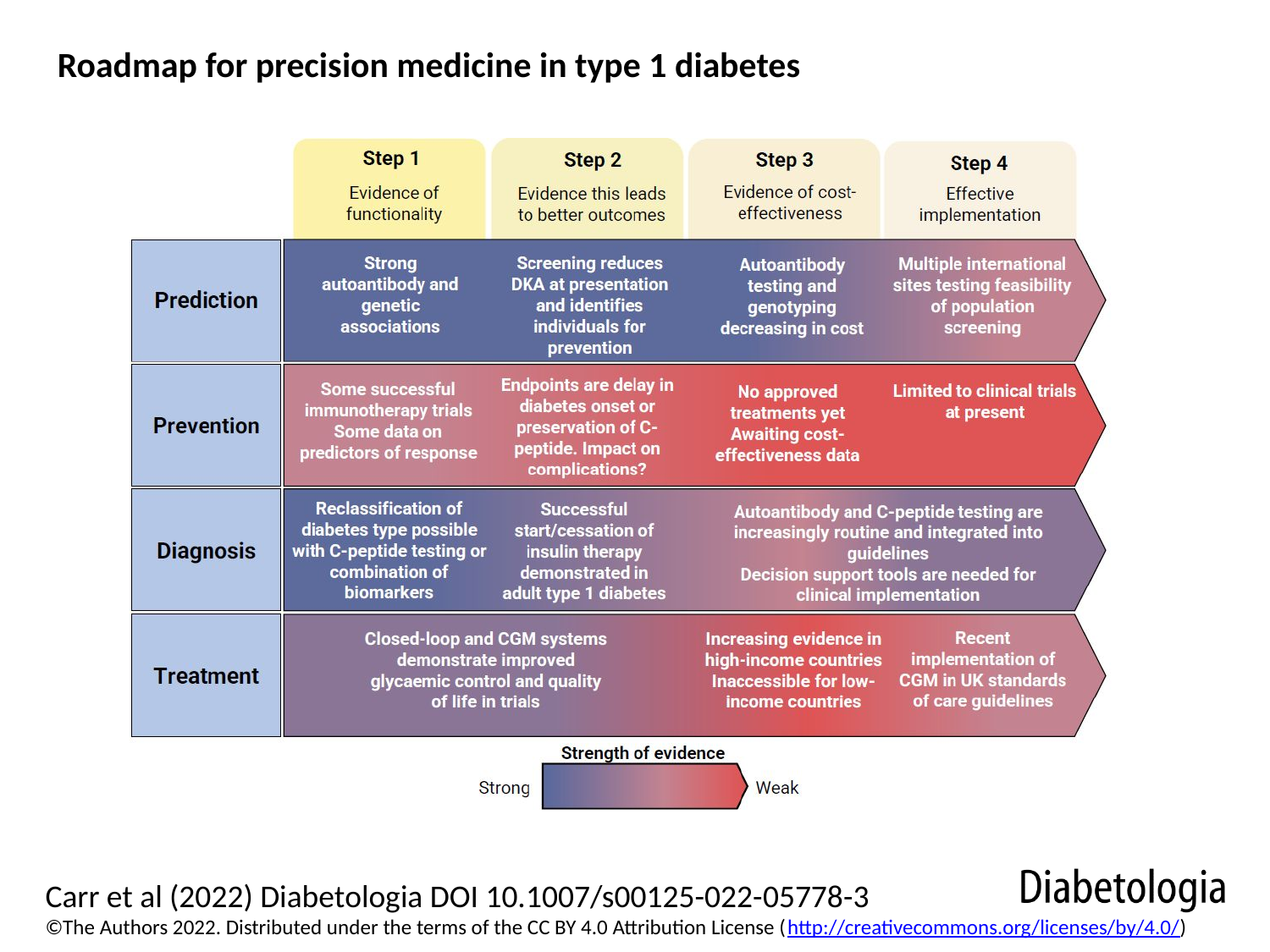

Roadmap for precision medicine in type 1 diabetes
Carr et al (2022) Diabetologia DOI 10.1007/s00125-022-05778-3
©The Authors 2022. Distributed under the terms of the CC BY 4.0 Attribution License (http://creativecommons.org/licenses/by/4.0/)
